# Syphilis Conveyed by Vaccination

**Published:** 1864-01

**Authors:** 


*Syphilis conveyed by Vaccination.*—Two instances of this have lately occurred in Paris. One of these was related to the Surgical Society of Paris by M. Chassaignac; and the other by M. Hirard to the Medical Society of Hospitals.

---

☞ Business letters to the Journal should be addressed to Drs. Miller & Ingals, Chicago, Ills., P. O. Drawer 5787. When money is received a receipt will be returned with the next number of the Journal, and subscribers failing to get such receipt will confer a favor by giving us notice of the omission.

